# B-cell-depleted patients with persistent SARS-CoV-2 infection: combination therapy or monotherapy? A real-world experience

**DOI:** 10.3389/fmed.2024.1344267

**Published:** 2024-02-29

**Authors:** Alessandra D’Abramo, Serena Vita, Alessia Beccacece, Assunta Navarra, Raffaella Pisapia, Francesco Maria Fusco, Giulia Matusali, Enrico Girardi, Fabrizio Maggi, Delia Goletti, Emanuele Nicastri

**Affiliations:** ^1^National Institute for Infectious Diseases “Lazzaro Spallanzani” IRCCS, Rome, Italy; ^2^Infectious Diseases Unit, "D. Cotugno" Hospital, AORN dei Colli, Naples, Italy

**Keywords:** persistent SARS-CoV-2 infection, B-cell depleted, combined therapy, antiviral, MoAbs

## Abstract

**Objectives:**

The aim of the study was to describe a cohort of B-cell-depleted immunocompromised (IC) patients with prolonged or relapsing COVID-19 treated with monotherapy or combination therapy.

**Methods:**

This is a multicenter observational retrospective study conducted on IC patients consecutively hospitalized with a prolonged or relapsing SARS-CoV-2 infection from November 2020 to January 2023. IC COVID-19 subjects were stratified according to the monotherapy or combination anti-SARS-CoV-2 therapy received.

**Results:**

Eighty-eight patients were enrolled, 19 under monotherapy and 69 under combination therapy. The study population had a history of immunosuppression (median of 2 B-cells/mm^3^, IQR 1–24 cells), and residual hypogammaglobulinemia was observed in 55 patients. A reduced length of hospitalization and time to negative SARS-CoV-2 molecular nasopharyngeal swab (NPS) in the combination versus monotherapy group was observed. In the univariable and multivariable analyses, the percentage change in the rate of days to NPS negativity showed a significant reduction in patients receiving combination therapy compared to those receiving monotherapy.

**Conclusion:**

In IC persistent COVID-19 patients, it is essential to explore new therapeutic strategies such as combination multi-target therapy (antiviral or double antiviral plus antibody-based therapies) to avoid persistent viral shedding and/or severe SARS-CoV-2 infection.

## Introduction

The World Health Organization (WHO) declared the end of the COVID-19 pandemic (1) on 5 May 2023, but the SARS-CoV-2 infection remains an ongoing problem in certain settings, especially in immunocompromised (IC) patients (2). These patients, particularly those with hematologic malignancies, are at an increased risk of SARS-CoV-2-associated morbidity and mortality due to the immunologic deficits that limit primary prevention, treatment, and clearance of the virus (3, 4). An impaired immune system impacts the natural course of the COVID-19 infection. Individuals with suppressed innate immunity may experience a higher incidence of infection, but they are able to control viral clearance. Patients with impaired adaptive cellular immunity have a high risk of death from acute infection (5, 6). Conversely, patients with impaired adaptive humoral immunity (i.e., B-cell malignancies and B-cell targeting therapy) have a high risk of prolonged viral shedding, viral rebound, and chronic infection. In this context, it is important to both identify these patients early and establish a timely and effective therapy (7, 8). The European and Italian Drug Agency both recommend the use of antivirals (remdesivir or nirmatrelvir/ritonavir) or monoclonal antibodies (MoAbs) against SARS-CoV-2 S-glycoprotein (sotrovimab or tixagevimab with cilgavimab) for primary prophylaxis or early treatment of COVID-19 patients at high risk of disease progression (9, 10). To date, there is no consensus on the clinical management of COVID-19 IC patients with prolonged viral persistence. Combination therapy (antiviral or double antiviral plus antibody-based therapeutics) is reported to be effective only in anecdotal cases (11–13). The aim of the study was to describe a cohort of B-cell-depleted IC patients with prolonged COVID-19 treated with monotherapy or combination therapy.

## Methods

This is a multicenter observational retrospective study conducted on IC patients consecutively hospitalized with prolonged SARS-CoV-2 infection at the Lazzaro Spallanzani National Institute for Infectious Diseases-IRCCS, Rome (INMI) and at the Ospedale Cotugno, Azienda Ospedaliera dei Colli, Naples, Italy from November 2020 to January 2023. This study defines the term “ImmunoCOVID,” which was approved by the INMI Spallanzani Ethics Committee (protocol number 315/2020–2021). Retrospective data from 20 patients had been previously reported (14, 15). Prolonged SARS-CoV-2 infection was defined by a SARS-CoV-2 positive real-time polymerase chain reaction (RT-PCR) in different biological samples, such as nasopharyngeal swabs (NPSs) or lower respiratory tract samples, with radiological and/or clinical evidence of infection after at least 21 days from the first positive SARS-CoV-2 NPS (16, 17). IC COVID-19 subjects were stratified according to the type of treatment received by patients on monotherapy or combination therapy. Combination therapy consists of antiviral plus antibody-based therapeutics (MoAbs or hyperimmune plasma donated by convalescent COVID-19 patients), double antivirals, or a triple combination (double antivirals plus MoAbs). Combination therapy was prescribed in an off-label protocol approved by the Hospital Pharmacist, Hospital Health Direction, and Italian Drug Agency. All patients signed informed consent for treatment and data collection. Demographic characteristics, medical history, clinical presentation, treatment, adverse drug reactions, virological, and clinical outcome (survival/death) at days 28 and 60 post-treatment were collected. In all patients, the SARS-CoV-2 diagnosis was made by RT-PCR and performed according to the laboratory workflow across various platforms, and the cycle threshold (CT) values were recorded when available. The tests were performed on NPS, spontaneous or induced sputum, or other lower respiratory tract samples. The date of infection diagnosis was considered the day of the first positive SARS-CoV-2 test (RT-PCR or antigen). The analyzed outcomes were time to virological response, defined as negative SARS-CoV-2 PCR in NPS or induced sputum after anti-SARS-CoV-2 treatment.

### Statistical analysis

The comparison of continuous data between the two groups of patients treated with monotherapy or combination antiviral therapy was analyzed with the Mann–Whitney test and summarized as the median and interquartile range (IQR). For categorical data, differences in treatment groups were assessed by the chi-square or Fisher exact test as appropriate. The truncated negative binomial regression models for univariable and multivariable analyses were used to study the days to viral clearance after treatment initiations as a function of treatment, demographic, and clinical characteristics. The minimum recorded number of days before viral clearance was 5 days; thus, the regression models were truncated at 4 days. Furthermore, all models were fitted with robust standard error estimates. The pandemic period and covariates with a *p*-value < 0.2 in the univariable model were entered in the final multivariable model. A two-tailed *p*-value < 0.05 was considered statistically significant. Statistical analyses were performed using Stata (StataCorp, 2021; Stata Statistical Software, Release 17; College Station, TX: StataCorp LLC).

## Results

### Patients

**Figure 1:**
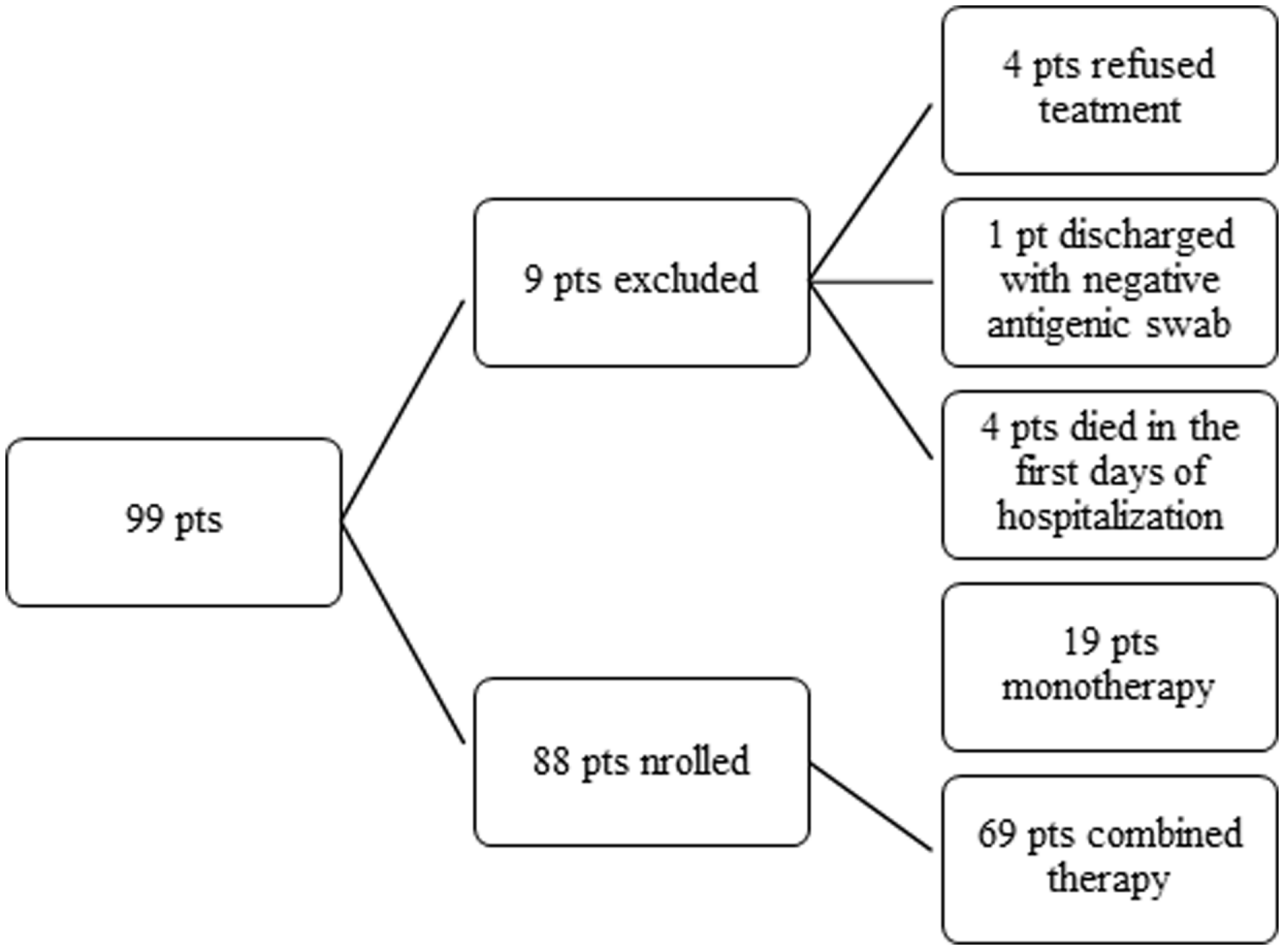
A flowchart of the study population.

From November 2020 to January 2023, 97 patients with B-cell depletion and SARS-CoV-2 infection were enrolled. A total of 9 patients were excluded (4 refused treatment, 1 was discharged with antigenic NPS, and 4 died in the first days of hospitalization). Finally, 88 patients were considered: 19 under antiviral monotherapy and 69 under combination antiviral therapy (Figure 1). Of the 88 patients, 66 patients (75.0%) had a hematological disorder, 10 patients (11.4%) had autoimmune diseases (psoriasis or rheumatoid arthritis), 9 patients (10.2%) had multiple sclerosis, and the remaining 3 patients (3.4%) had a kidney transplantation (2 subjects) or HIV infection. The study population had a history of immunosuppression (a median of 2 B-cells/mm^3^, IQR 1–24 cells) with residual hypogammaglobulinemia in 55 patients (62.5%); 11 hematologic patients had gamma globulin normal values, of which 7 had non-Hodgkin’s lymphoma with B-cell depletion, whereas the remaining 4 patients had chronic lymphatic leukemia under tyrosine kinase inhibitor (TKI) treatment. A total of 66 patients (75.9%) had completed a full vaccination course with an anti-SARS-CoV-2 mRNA vaccine and received at least one booster dose. Four patients had received long-acting human MoAbs (tixagevimab/cilgavimab) as pre-exposure prophylaxis. Early treatment was administered within 5 days of symptom onset in 17 patients (19.3%). Of the 17 patients, 3 were treated with remdesivir, 4 with nirmatrelvir/ritonavir, 5 with molnupiravir, and 5 with MoAbs (sotrovimab, casirivimab-imdevimab, and tixagevimab/cilgavimab). At hospital admission, positive anti-SARS-CoV-2 serology was reported in 9 patients (10.2%) (Table 1). In 40 enrolled patients (45.4%), the SARS-CoV-2 variants of concern (VoCs) were identified: 2 cases had an alpha VoC and 38 patients had several omicron VoCs, according to the ongoing epidemiology in Italy (Supplementary Table S1).

**Table 1:** Characteristics of the enrolled patients according to SARS-CoV-2 treatment.

Patients’ characteristics		Overall	SARS-CoV-2 treatment		*p*-value
			Monotherapy	Combination therapy	
		*n* (%)	*n* (%)	*n* (%)	
Total		88	19 (21.6)	69 (78.4)	
Age in years	Median (IQR)	63 (54–76)	61 (51–76)	64 (55–76)	0.348
Sex	Female	41 (46.6)	10 (52.6)	31 (44.9)	0.551
Vaccination status	Vaccinated	66 (75.9)	10 (52.6)	56 (82.4)	**0.007**
Underlying diseases	Autoimmune diseases	9 (10.2)	2 (10.5)	7 (10.1)	0.332
	Hematological diseases	66 (75)	13 (68.4)	53 (76.8)	
	Neurological diseases	10 (11.4)	2 (10.5)	8 (11.6)	
	Others (transplantation/HIV)	3 (3.4)	2 (10.5)	1 (1.5)	
Immunosuppressive treatments	None	22 (25)	4 (21.1)	18 (26.1)	0.258
	Yes, Anti-CD20	50 (56.8)	9 (47.4)	41 (59.4)	
	Yes, others	16 (18.2)	6 (31.6)	10 (14.5)	
Chronic steroid treatment	No	64 (72.7)	13 (68.4)	51 (73.9)	0.455
	Yes	24 (27.3)	6 (31.6)	18 (26.1)	0.388
Comorbidities, detail	Diabetes mellitus	8 (9.1)	1 (5.3)	7 (10.1)	1.000
	Heart disease	21 (23.9)	4 (21.1)	17 (24.6)	1.000
	Hypertension	36 (40.9)	10 (52.6)	26 (37.7)	0.241
	Respiratory disease	12 (13.6)	3 (15.8)	9 (13)	0.717
	Kidney disease	7 (8)	3 (15.8)	4 (5.8)	0.169
	Hepatic disease	3 (3.4)	0 (0)	3 (4.4)	1.000
	BMI > 30	8 (9.1)	0 (0)	8 (11.6)	0.193
Pandemic period, *n* (%)					0.013
	Before 01/01/2022	26 (29.6)	10 (52.6)	16 (23.2)	
	After 01/01/2022	62 (70.4)	9 (47.4)	53 (76.8)	
Total Lymphocytes at baseline	Median (IQR)	0.83 (0.42–1.28)	0.64 (0.4–1.32)	0.87 (0.47–1.26)	0.574
Lymphocytes CD20 at baseline	Median (IQR)	2 (1–24)	5 (1–129)	2 (1–21)	0.086
Hypogammaglobulinemia	No	33 (37.5)	8 (42.1)	25 (36.2)	0.558
	Yes	55 (62.5)	11 (57.9)	44 (63.8)	0.633
Days from symptoms onset to hospitalization	Median (IQR)	21 (7–40)	10 (3–78)	22.5 (8.5–37.5)	0.420
Pneumonia	No	7 (7.9)	2 (10.5)	5 (7.2)	0.544
	Yes	81 (92.1)	17 (89.5)	64 (92.8)	0.641
P/F at admission	Median (IQR)	352 (286–419)	332 (286–390)	357 (286–457)	0.460
Days from positivity to therapy initiation	Median (IQR)	19 (5.5–44)	14 (3–48)	21 (8–36)	0.549
Length of hospitalization (days)	Median (IQR)	22.5 (16.5–36.5)	30 (21–48)	21 (14.5–32.5)	**0.047**
Viral Shedding (days)	Median (IQR)	48 (32–85)	40 (26–60)	23 (13–32)	**0.002**

All statistically significant *p* values in bold.

### Clinical features and outcome

All patients were symptomatic for the SARS-CoV-2 infection at the time of treatment. Of these patients, 81 (92.1%) had interstitial pneumonia, of which 11 patients (13.6%) showed mild symptoms, and 70 patients (86.4%) were in need of oxygen therapy. A total of 23 patients (26.1%) required continuous positive airway pressure or non-invasive ventilation; of them, three were admitted to the intensive care unit (ICU). A total of 19 (21.6%) patients received monotherapy, whereas 69 (78.4%) patients received combination therapy (Supplementary Table S2). The combination therapy consisted of intravenous antiviral (remdesivir 200 mg on day 1 followed by 100 mg every day) plus antibody-based therapeutics (45 pts): 1 unit of 250 mL of hyperimmune convalescent plasma (1:320 neutralizing Ab titer against SARS-CoV-2 spike glycoprotein) every 48 h × 3 doses in 5 patients or MoAbs in 40 patients: sotrovimab (20 cases), casirivimab-imdevimab (10 cases), and tixagevimab/cilgavimab (10 cases). The double antiviral regimen included a combination of intravenous remdesivir plus oral nirmatrelvir/ritonavir (300/100 mg twice per day), and the triple combination regimen included a combination of the previously mentioned double antiviral regimen plus sotrovimab (six cases) or tixagevimab/cilgavimab (seven cases) (Supplementary Table S2). Overall, the median time to hospitalization from symptom onset was 21 (IQR 7–40) days, the median duration of SARS-CoV-2 viral shedding was 50 (IQR 32–83.5) days, and the median length of hospital stay (LOS) was 22.5 days (IQR 16.5–36.5). The median duration of antiviral therapy in patients receiving combination therapy was 10 days, with a maximum in some cases of up to 30 consecutive days. No severe adverse events were reported, whereas side effects were mild (taste disturbances and nausea), and neither treatment modification nor symptomatic therapy was needed.

### Monotherapy versus combination therapy

Stratifying patients according to treatment, the median time to hospitalization from symptom onset was 10 (IQR 3–78) and 22.5 (8.5–37.5) days in the monotherapy group versus combination therapy, respectively (*p* = 0.420), whereas the median time from positive NPS to therapy initiation was 14 days (3–48) versus 21 days (8–36), respectively (*p* = 0.549) (Table 1).

A significantly reduced LOS and time to negative SARS-CoV-2 molecular NPS in the combination group versus monotherapy group were observed (21 vs. 30 days for LOS, *p* = 0.047 and 23 vs. 40 days for negative NPS, *p* = 0.002, respectively).

In the univariable analysis, the percentage change in the rate of days to NPS negativity showed a significant reduction of 28% in patients receiving combination therapy compared to those receiving monotherapy (incidence rate ratio [IRR]: 0.72, 95% confidence interval [CI]: 0.53–0.98, *p* = 0.035). This reduction was confirmed on the multivariable analysis after adjustment for pandemic period, age, and weeks elapsed from positivity to start of therapy (IRR: 0.71, 95% CI: 0.51–1.00, *p* = 0.050). Furthermore, in both univariable and multivariable analyses, patients with a hematological disease demonstrated increased time to NPS negativity (IRR: 1.55, 95%CI: 1.08–2.21, *p* = 0.016 and IRR: 1.66, 95% CI: 1.08–2.53, *p* = 0.020, respectively) (Table 2).

**Table 2:** Univariable and multivariable analyses.

	Truncated negative binomial regression*					
	Univariable			Multivariable		
Characteristics	IRR**	95%CI	*p*	IRR	95%CI	*p*
**SARS-CoV-2 treatment**						
Monotherapy	Ref.			Ref.		
Combination therapy	0.72	0.53–0.98	**0.035**	0.71	0.51–1	**0.050**
**Age**	1.07	0.98–1.17	0.115	1.00	0.9–1.12	0.970
**Sex**						
Male	Ref.					
Female	1.07	0.78–1.47	0.657			
**Pandemic period**						
Before 01 January 2022	Ref.			Ref.		
After 01 January 2022	0.86	0.63–1.18	0.346	1.13	0.74–1.72	0.570
**Weeks from positivity to therapy initiation**	0.98	0.94–1.01	0.140	1.00	0.99–1	0.100
**Vaccination status**						
Unvaccinated	Ref.			Ref.		
Vaccinated (at least two doses)	0.75	0.54–1.06	0.101	0.74	0.46–1.19	0.221
**Type of immune disease**						
Autoimmune disease	0.64	0.36–1.14	0.132			
Hematological disease	Ref.					
Neurological disease	0.57	0.36–0.91	0.019			
Others (transplantation/HIV)	0.89	0.48–1.64	0.713			
**Hematological disease versus other**	1.55	1.08–2.21	**0.016**	1.66	1.08–2.53	**0.020**
**Immunosuppressive treatments**						
No	Ref.					
Yes	1.10	0.82–1.47	0.515			
**Presence of comorbidities**						
No	Ref.					
Yes	0.95	0.69–1.32	0.776			
**Hypogammaglobulinemia**						
No	Ref.					
Yes	0.93	0.68–1.25	0.616			
ND	0.83	0.43–1.62	0.587			
**CD20 at baseline**						
<2	Ref.					
≥2	1.06	0.76–1.49	0.725			
**Lymphocytes at baseline** (square root transformation)	0.97	0.92–1.02	0.211			
**P/F at admission** (for increment of 100)	0.90	0.76–1.06	0.205			
**Pneumonia**						
No	Ref.					
Yes	1.41	0.67–3.00	0.369			

The results from the truncated negative binomial model.

*Truncated at day 4. IRR, incidence rate ratio; CI, confidence interval.

All statistically significant *p* values in bold.

## Discussion

The SARS-CoV-2 infection remains an ongoing clinical challenge in certain settings, especially in IC patients. These patients, particularly those with hematological malignancies, are at an increased risk of morbidity (50–80%) and mortality (20–40%) associated with SARS-CoV-2 infection (4, 18).

An impaired immune system impacts the natural course of COVID-19 infection, and in particular, patients with B-cell depletion have a high risk of prolonged viral shedding, viral rebound, and chronic infection (6).

To our knowledge, the cohort used in this study is the largest of B-cell-depleted patients with a prolonged SARS-CoV-2 infection comparing monotherapy and combination therapy with a case fatality rate of 4%.

IC individuals have been under-represented in previous registration of randomized clinical trials but are likely to be over-represented among currently hospitalized patients with severe or persisting symptoms due to SARS-CoV-2, as they have impaired responses to vaccination and/or previous natural infection (19–21).

To date, there is no therapeutic consensus in IC patients with prolonged persistence of SARS-CoV-2 infection; combination antiviral therapy has been reported to be safe and effective in anecdotal cases only (16, 17, 22–24). In our cohort, we used different regimens of antiviral monotherapy (mostly in the early phase of the pandemic) and combination therapy, always on off-label prescription. In line with the literature, all prescribed regimens appear to be a safe and effective drug strategy for obtaining a virological and clinical cure. Briefly, 88 IC COVID-19 patients were enrolled and 69 of them, who were treated with combination therapy, experienced a significant reduction in both length of hospitalization and time to negative SARS-CoV-2 molecular NPS compared to those in antiviral monotherapy.

In our cohort, the presence of a hematological disease was associated with a prolonged positive NPS. In the univariable analysis, the percentage change in the rate of days to NPS negativity showed a significant reduction in patients receiving combination therapy compared to those receiving monotherapy, and this reduction was confirmed in the multivariable analysis after adjustment for pandemic period, age, and weeks elapsed from positivity to the start of therapy.

In our population, the majority of the patients in combination therapy were fully vaccinated compared to half of the patients in monotherapy. Controversial data are available on the impact of vaccination status on the duration of viral shedding in IC patients (25, 26), and our results are not likely to suggest a direct correlation between them. Obviously, SARS-CoV-2 vaccination prevents severe disease in the general population, but the COVID-19 risk remained elevated across IC groups in terms of COVID-19 hospitalization, ICU admissions, and mortality. However, the effectiveness of SARS-CoV-2 vaccination differs between individuals depending on the underlying disease and immunosuppressive agents (27). Moreover, passive immunotherapy (MoAbs and/or hyperimmune convalescent plasma) against the SARS-CoV-2 infection represents the main prophylactic and therapeutic options as a source of exogenous specific antibodies in IC patients with primary or secondary humoral disorders.

Limitations of the study. First, this is a retrospective observational study conducted in two of the main health centers dedicated to the clinical management of infectious diseases in Italy on a relatively small population. The limited number of enrolments could influence the generalization of our results to a larger population of IC patients with COVID-19. However, our results are consistent with data from previous studies and report the largest cohort of patients with prolonged infections treated with unlicensed antiviral agents. Second, the heterogeneity of the treatment protocol is due to different epidemic phases of patient enrollment and to the different availability of full effective MoAbs and antivirals from November 2020 to January 2023. In this time period, SARS-CoV-2 evolved from the S/L (wild-type) lineage to the Omicron lineage, with a drastic change in virulence and infectivity/transmissibility in the general population but always maintained high morbidity and mortality in IC COVID patients. Third, full identifications of SARS-CoV-2 VoC were performed in a minority of the study populations, and we could indirectly assume the viral circulation on community epidemiologic data only.

Strengths of the study: Our cohort also has some strengths. First, this is the largest cohort of patients with prolonged SARS-CoV-2 infection with B-cell depletion comparing monotherapy and combination therapy. To date, there is no therapeutic consensus in IC patients with prolonged SARS-CoV-2 persistence, and most of the registered comparative clinical trials on the clinical management of COVID-19 have a limited number of COVID-19 patients. Combination therapy in IC COVID-19 patients is not standardized, despite growing scientific evidence of virological and clinical efficacy; cohort studies in this setting may be a driver of building preliminary evidence for future comparative studies. Second, the risk of prolonged viral persistence in IC COVID-19 patients appears to be related to active hematological disease, which delays access to the cure for the underlying disease.

## Conclusion

Innovative therapeutic approaches such as combination multi-target therapy (including antiviral and antibody-based therapies) are needed in IC patients with persistent COVID-19. These approaches are likely to prevent prolonged viral shedding and severe SARS-CoV-2 infections, resuming the cure for underlying diseases and increasing the quality of life of IC COVID-19 patients.

## Data availability statement

The original contributions presented in the study are included in the article/Supplementary material, further inquiries can be directed to the corresponding author.

## Ethics statement

This study entitled ‘ImmunoCOVID’ was approved by the INMI Spallanzani Ethics Committee (protocol number 315/2020-2021). The study was conducted in accordance with local legislation and institutional requirements. Participants provided written informed consent to participate in this study. Written informed consent was obtained from the individual(s) for the publication of any potentially identifiable images or data included in this article.

## Author contributions

AD’A: Conceptualization, Data curation, Supervision, Writing – original draft, Writing – review & editing. SV: Validation, Writing – original draft, Writing – review & editing. AB: Data curation, Investigation, Writing – review & editing. AN: Formal Analysis, Methodology, Writing – review & editing. RP: Investigation, Writing – review & editing. FF: Investigation, Writing – review & editing. GM: Investigation, Methodology, Writing – review & editing. EG: Supervision, Validation, Writing – review & editing. FM: Investigation, Supervision, Validation, Writing – review & editing. DG: Supervision, Validation, Writing – review & editing. EN: Funding acquisition, Supervision, Validation, Writing – review & editing.

## ImmunoCOVID team

Tommaso Ascoli Bartoli, Nazario Bevilacqua, Angela Corpolongo, Patrizia De Marco, Maria Letizia Giancola, Gaetano Maffongelli, Andrea Mariano, Laura Scorzolini, Claudia Palazzolo, Silvia Rosati, Virginia Tomassi, Francesca Faraglia, Lavinia Fabeni, Martina Rueca, Silvia Meschi, and Cesare Ernesto Maria Gruber.
