# Supplementary material for: B-cell-depleted patients with persistent SARS-CoV-2 infection: combination therapy or monotherapy? A real-world experience
Source: Front Med (Lausanne). 2024 Feb 29;11:1344267. doi: 10.3389/fmed.2024.1344267 (PMC10937561; doi:10.3389/fmed.2024.1344267)
Supplement: Supplementary file 1 [file Table_1.DOCX]

| Table1. SARS-CoV-2 variants in the period study |  |  |
| --- | --- | --- |
| Alfa (n,%) | 2 | 2.1 |
| Omicron (n,%) | 36 | 37.1 |
| BA.1 | 6 |  |
| BA.2 | 15 |  |
| BA.5 | 6 |  |
| BQ.1 | 4 |  |
| BF | 2 |  |
| Other (n,%) | 2 | 2.1 |
| Undetermined (n,%) | 57 | 58.7 |
