# Supplementary material for: B-cell-depleted patients with persistent SARS-CoV-2 infection: combination therapy or monotherapy? A real-world experience
Source: Front Med (Lausanne). 2024 Feb 29;11:1344267. doi: 10.3389/fmed.2024.1344267 (PMC10937561; doi:10.3389/fmed.2024.1344267)
Supplement: Supplementary file 2 [file Table_2.DOCX]

| Table2. Therapeutic schemes performed in the study population |  |  |
| --- | --- | --- |
| MONOTHERAPY (n,%) | 19 | 21.6 |
| Antiviral (RDV) | 10 | 52.6 |
| Plasma or MoAbs | 9 | 26.7 |
| MoAbs (casirivimab-imdevimab /Sotrovimab/ Bamlanivimab/etesevimab) | 6 | 66.6 |
| Plasma | 3 | 33.4 |
| COMBINED THERAPY (n,%) | 69 | 78.4 |
| Antiviral+ Plasma or MoAbs | 45 | 65.2 |
| RDV+ Casirivimab-imdevimab | 8 | 17.8 |
| RDV+ Sotrovimab | 20 | 44.5 |
| RDV+ Tixagevimab/cilgavimab | 11 | 24.4 |
| RDV+ Bamlanivimab/etesevimab | 1 | 2.2 |
| RDV+ Plasma | 5 | 11.1 |
| Double antiviral |  |  |
| RDV+nirmatrelvir/ritonavir | 11 | 15.9 |
| Triple combination | 13 | 18.9 |
| RDV+nirmatrelvir/ritonavir + Sotrovimab | 7 | 53.8 |
| RDV+nirmatrelvir/ritonavir+ Tixagevimab/cilgavimab | 6 | 46.2 |
